# AI Health Coach Prototype and Personalized Pathophysiological Hypotheses Based on an N-of-1 Big Data Study of Non-specific Musculoskeletal Chronic Pain

**DOI:** 10.7759/cureus.117549

**Published:** 2026-10-06

**Authors:** Olivier Mirat

**Affiliations:** 1 Health Informatics, Independent Researcher, Lyon, FRA

**Keywords:** acute:chronic workload ratio, artificial intelligence (ai), central sensitization syndromes, chronic musculoskeletal pain, digital phenotyping, just-in-time adaptive interventions, n-of-1 trial, personalized medicine (pm), random forest, wearable technologies

## Abstract

Background

Modern healthcare is shifting towards predictive, preventive, personalized, and participatory medicine. While machine learning (ML) forecasting has seen success in episodic conditions, translating these results to non-specific musculoskeletal chronic pain is challenging due to the logistical barriers of collecting high-density, continuous longitudinal data across large cohorts. Therefore, this study leverages a high-density n-of-1 dataset to (i) generate personalized data-driven hypotheses about symptom-stressor associations and pathophysiology, and (ii) explore the feasibility of a prospective AI prediction tool.

Methods

A longitudinal n-of-1 observational study was conducted on a single adult male with mechanically exacerbated non-specific musculoskeletal chronic pain. Over 10 years (utilizing a 28-month core analysis window), continuous subjective daily pain logging was combined with high-resolution physiological monitoring via a commercial wearable ecosystem. Feature engineering utilized the Acute:Chronic Workload Ratio (ACWR) framework to quantify physiological stressors across three pain regions (face, knee, and arm). Data were analyzed using Pearson correlations, and random forest classifiers were trained to generate a daily, actionable AI-generated traffic light flare warning system.

Results

Both correlation analyses and single-stressor random forest models revealed that stressors applying direct mechanical load to a specific region (e.g., computer use for face pain, step count for knee pain) were most strongly associated with symptom flares, outperforming systemic variables like sleep or mood. In the held-out test set, the AI coach prototype achieved receiver operating characteristic (ROC)-area under the curve (AUC) values of 0.93 (face), 0.78 (knee), and 0.82 (arm) between clearly high and low pain days and correctly flagged each region's single most severe flare with a red or orange warning before its peak. Since only one such flare occurred per region, this should be read as an illustrative result rather than an estimate of general reliability; a fuller, more mixed picture across all flare events is reported in Results. Furthermore, evaluation on a subsequent follow-up dataset held out during previous training and tuning decisions showed decreased alert performance, reflecting real-world deployment complexities or genuine instability in the learned stressor-pain relationships. Regardless, prediction probabilities still rose before flares. Finally, counterfactual analysis of an ergonomic intervention through AI predictions led to results consistent with, but not proof of, ergonomics-related pain reduction.

Conclusions

This proof-of-concept illustrates how an automated n-of-1 pipeline can serve a dual function. First, combining linear correlation with non-linear ML on ACWR-engineered features, this study identifies associations between regional mechanical load and pain symptoms that are consistent with, but do not establish, a load-related pain mechanism such as bottom-up central sensitization, and points toward the envelope of function as a candidate therapeutic framework. Second, it illustrates the potential to translate these associations into a foundational just-in-time adaptive intervention system, empowering patients to proactively manage daily mechanical loads. Future integration of objective biological markers may further transition such tools from associative forecasting to true causal understanding.

## Introduction

The paradigm of modern healthcare is shifting from episodic, reactive care toward predictive, preventive, personalized, and participatory medicine, or the '4P' framework [1]. This transition is critical because traditional reactive models intervene only after symptoms have escalated, at which point the condition becomes significantly harder to manage. In the context of chronic pain, this delayed intervention not only prolongs patient suffering but also drives massive and unsustainable healthcare costs [2]. To overcome these limitations, the 4P approach aims to enable preemptive action by leveraging continuous data to anticipate exacerbations and tailor interventions to an individual's unique biological profile. The integration of wearable technology and digital phenotyping may offer a practical mechanism to achieve this, enabling the detection of physiological precursors to symptom flares before they manifest clinically.

The application of machine learning (ML) to precision medicine has been widely explored, yet a distinction must be drawn between retrospective association studies and prospective forecasting systems [3]. The first category of studies focuses on establishing biological plausibility. For example, recent work has demonstrated statistically significant associations between physiological metrics (such as autonomic heart rate markers) and symptom flares in conditions like rheumatoid arthritis [4] and fibromyalgia [5], as well as between musculoskeletal load and symptoms in chronic non-specific musculoskeletal pain [6]. While these studies confirm that physiological signals often precede symptoms, they rely on retrospective statistical frameworks (such as mixed-effect models, multilevel regression, or longitudinal trend analyses) to quantify explanatory associations. Consequently, they stop short of developing prospective algorithms capable of generating actionable, real-time predictions for individual patients.

The second category attempts to build functional forecasting engines. In neurology, seizure prediction systems have achieved robust validation [7]. Similar efforts have extended to other episodic conditions like asthma [8] and migraines, where ML models utilizing preictal wearable data and digital diaries have successfully forecast subsequent-day attacks [9], guiding early intervention. Recently, predictive modeling has begun expanding into the chronic pain space; for example, short-term cohort studies have demonstrated that ML can predict acute pain spikes in patients with co-occurring opioid use disorder [10]. However, translating cohort-based models to complex, chronic, non-specific musculoskeletal pain remains a challenge. Accurate prediction in this domain requires capturing continuous, high-density physiological and mechanical data over extended periods. Collecting this caliber of long-term, multi-modal data across a large cohort is logistically prohibitive and prone to high dropout rates [11]. Consequently, before scaling to broader populations, establishing model feasibility through a rigorous n-of-1 deep-phenotyping approach serves as a necessary first step.

Non-specific chronic pain conditions present a distinct management challenge; unlike inflammatory diseases with specific biomarkers, the underlying biological mechanisms of non-specific pain are often unknown [12]. Because the patient in this n-of-1 study presented with symptoms that were highly mechanically exacerbated by daily physical activities (previously detailed as participant #1 in [6]), the predictive modeling in this study focuses on quantifying the impact of these mechanical loads on symptoms while also incorporating systemic variables such as mood or sleep quality.

The objective of this study is two-fold. The first aim is to explore the possibility of identifying correlations between symptoms and stressors in a high-density n-of-1 personal dataset, and to use those correlations to generate personalized pathophysiological hypotheses about the patient. The second aim is to develop and evaluate a prospective, personalized AI decision support system for a single patient with chronic non-specific musculoskeletal pain (an n-of-1 design), implemented as a daily 'traffic light' (green/orange/red) early warning signal. This system directly alerts the patient to their personalized risk of an impending flare based on their cumulative physiological load over the past weeks and their planned activities for the current day, serving as a basis for just-in-time adaptive interventions, which are designed to deliver real-time, context-aware behavioral guidance [13]. A secondary, exploratory aim is to examine whether such a system can also help evaluate the impact of a lifestyle intervention on an individual's risk profile. 

Regarding the personalized prediction system, this initial proof of concept focuses exclusively on predicting symptom flares rather than forecasting minor daily fluctuations in baseline pain. Indeed, the impact of these exacerbations extends far beyond the acute event itself: while subjective pain ratings may return to baseline within a matter of days, the patient’s physical activity and load capacity often remain significantly suppressed for weeks as they instinctively avoid triggering the lingering sensitivity, leading to a restrictive cycle of activity and rest [6,14]. Furthermore, from an ML perspective, focusing on these major exacerbations offers a much higher signal-to-noise ratio. Predicting distinct, high-intensity flare events is a much more tractable first task than predicting subtle daily variations, making it an ideal, clinically relevant target for establishing foundational model feasibility.

## Materials and methods

Participant and study design

This study employed a longitudinal n-of-1 observational design following a single subject: an adult male with a documented history of mild to moderate chronic non-specific musculoskeletal pain. The participant is the same individual characterized as participant #1 in the author's previous study [6], who is also the author of this paper. The subject's pain was primarily exacerbated by mechanical load, characterized by fluctuating periods of remission and flares that had proven resistant to standard physical therapy. Initial pain onset for each affected body region typically followed periods of elevated physical exertion or sustained postural strain (e.g., prolonged computer use). Major structural pathologies and systemic inflammatory conditions (such as rheumatoid arthritis) were previously ruled out via imaging and blood tests, and the subject was otherwise healthy.

Compared to the previous study, new data were collected continuously over a 28-month period from mid-May 2023 to mid-September 2025, which will be referred to as the 'new dataset.' This period was defined by the transition to a single, continuously worn wearable device (the Garmin Vivosmart 5; Garmin International, Olathe, KS, USA), which, unlike the mix of devices used previously, provided uninterrupted, higher-precision tracking, including consistent monitoring during swimming and surfing for the first time, together with the introduction of daily sleep score and mobile-phone-use logging. The subject was 34 years old at the onset of this new data collection period, and his BMI remained stable between 20 and 24 across all study periods. The patient’s pain was categorized into three primary anatomical regions: the face, knee, and arm.

Historical data from early January 2016 to late April 2023 will be referred to as the 'old dataset.' Of this period, data through March 2022 were previously analyzed, using linear statistical methods, in the author's prior study [6]; the remainder of the old dataset (March 2022 to April 2023), along with the entirety of the new dataset and follow-up dataset described below, has not been previously reported. The overlapping portion is reused here only to evaluate the present study's Acute:Chronic Workload Ratio (ACWR)-based [15] random forest pipeline, an analysis not applied to it in the prior study; no observations from this overlapping period are included in the present study's own statistical analyses. While the two studies' conclusions broadly converge, they do so via independent, non-overlapping methods. Finally, a follow-up dataset recorded from mid-September 2025 to mid-July 2026 was utilized. This dataset shares the same structure as the new dataset but was reserved as a held-out evaluation set, distinct from the internal test set described below. The AI coach prototype was primarily developed between January and March 2026 using only the new dataset.

The aim of the follow-up dataset was to evaluate the frozen model's real-world, prospective performance, a role the internal test set cannot fully substitute for. This separation was not fully maintained for the knee-pain prediction model. Its cycling-related stressor was initially 'number of calories while cycling'; after the follow-up dataset had already been used to evaluate this configuration, this stressor was replaced in July-August 2026 with 'cycling cumulative bpm > 110,' with no other change to the knee-pain model. This updated feature is what is reported for knee pain throughout this paper; the face and arm models were not affected. Because this substitution was informed by the follow-up dataset's own evaluation outcome, the follow-up performance reported for knee pain reflects a partly retrospective adjustment rather than a fully prospective test. Separately, the multistep residual removal Pearson correlation, the load irregularity analysis, and the single-stressor comparisons involving 'cycling cumulative bpm > 110' were also conceived and conducted in July-August 2026, using the new dataset only, and are reported as exploratory and post-hoc. None of these analyses fed back into the frozen prediction pipeline evaluated above.

Data acquisition and processing

Data collection involved combining subjective symptom logging with high-resolution objective physiological monitoring. Subjective measures were consistent with the protocol established in the author's previous work [6]; the participant logged pain intensity daily on a scale from 0 to 10, developed and applied consistently by the participant throughout the study period. This scale was purpose-built for continuous daily self-tracking rather than adapted from an instrument designed for periodic clinical assessment. Qualitative descriptors were assigned at the following score thresholds: 1 = almost imperceivable; 2 = could feel it; 3 = uncomfortable; 3.2 = uncomfortable+; 3.4 = uncomfortable++; 3.6 = very uncomfortable; 3.8 = very uncomfortable+; 4.5 = painful; 6 = very painful; 8 = extremely painful; 9.5 = worst pain ever. Anchor density is highest between 3.0 and 3.8, reflecting deliberately finer resolution in the range most relevant to the quantile-based classification described in the 'ML architecture' and 'evaluation metric' subsections below. General mood was also logged daily using one of eight categories: (i) really good, (ii) good, (iii) fine, (iv) variable but mostly good, (v) ok, but also not really that great, (vi) variable but mostly not great, (vii) tired, or (viii) depressed, with each category optionally further modified by a '+' or '-' sign. Additionally, specific physical activities (e.g., walking, cycling, climbing) were logged with timestamped start and end times to allow for precise temporal mapping, and some values (such as time spent driving or riding in a car or number of vertical meters of rock climbing) were manually estimated by the participant every evening, at the same time as pain and mood were logged, based on the participant's recall at the end of the day, rather than a record made at the time. 

For this new study phase (i.e., the new dataset), objective measures (physiological and kinematic) were collected with the Garmin Vivosmart 5 (Garmin International, Olathe, KS, USA), which replaced the consumer-grade activity trackers used previously, such as Fitbit (Fitbit Inc., San Francisco, CA, USA) and Basis Peak (Basis Science Inc., San Francisco, CA, USA). Fine-grained intra-day time series data were extracted, and raw data streams (including the number of heartbeats per minute and calories burned) were synchronized with the subjective activity logs. Additionally, daily aggregated physiological metrics, specifically overnight sleep scores, were recorded via the wearable device. This granular approach allowed for the isolation of physiological loads specific to distinct activity windows, enabling a more precise quantification of load on different body regions. Time spent on a computer and mouse/keyboard click counts were logged automatically via two dedicated computer-monitoring applications, ManicTime (Finkit d.o.o., Ljubljana, SVN) for time spent on the computer and WhatPulse (Enschede, OV, NLD) for the number of mouse/keyboard clicks.

For the new dataset (and for the follow-up dataset), the specific stressors listed below were used for each ML predictor, calculated on a daily basis. Face pain: time spent on a computer; time spent driving a car; time spent riding in a car, bus, or train (not driving); time spent using a mobile phone. Knee pain: number of steps taken; time spent on a computer; time spent driving a car; cumulative heartbeats above 110 bpm during cycling. Arm pain: number of computer mouse clicks and keyboard strokes; cumulative heartbeats above 110 bpm during upper-body activities (e.g., swimming, rock climbing, or surfing); cumulative heartbeats above 110 bpm during surfing; rock climbing maximum route grade.

For the old dataset, the same stressors were utilized where possible; however, these data lacked the fine-grained resolution of the new dataset. Specifically, they did not track time spent using a mobile phone or riding in a vehicle (as a passenger). Furthermore, physiological metrics were less precise, lacking active calorie and localized heart rate tracking during specific activities. Consequently, activities like surfing were recorded as highly approximate binary variables, swimming was based on estimated distance, and cycling relied on artifactual step counts, which also occasionally persisted during scooter riding and rock climbing despite removal efforts. Given the 7.5-year span of this dataset and the sequential use of three different fitness tracker models (Basis Peak, Fitbit Charge HR, and Fitbit Charge 5), all hardware data were normalized prior to analysis, and a 270-day rolling min-max scaler was applied to every stressor. Step count required additional harmonization across sources, using Fitbit-recorded values as the reference scale. Alternative sources available when Fitbit coverage was absent (the Basis Peak device, the Moves smartphone app, Google Fit, depending on the period) were calibrated against this reference wherever the two overlapped in time. This introduces further, unquantified uncertainty into step-count-derived stressors for the old dataset beyond the resolution limitations already noted. Because of this uncertainty and lower resolution, model performance and correlations observed in this dataset are expected to be weaker.

Ethical statement

Institutional Review Board (IRB) approval was not sought for this study prior to data collection or analysis. The following features of the study are relevant to its ethical evaluation: the sole human subject is the author himself, who provided full, autonomous, informed consent to the collection, analysis, and publication of his own data; no third-party data of any kind, identifiable or otherwise, is included in this study; and the study involved no intervention, procedure, or exposure beyond the subject's normal daily activities and the use of a commercial consumer wearable device. For context, the use of self-collected, auto-observational personal data for analysis and publication has precedent in the literature published without formal IRB oversight, including the use of wearable sensors to track personal environmental exposure [16], the longitudinal analysis of dream logs [17], and the multi-year tracking of biometric cardiovascular trends [18].

Data preprocessing and feature engineering 

Feature engineering was grounded in the ACWR model [15], a sports-medicine framework relating injury risk to sudden load spikes relative to accumulated tolerance, applied here on an exploratory basis, under the hypothesis that an analogous load-versus-tissue-capacity relationship also underlies this patient's chronic pain flares. This approach aligns with the tissue homeostasis perspective [19] and the short- and long-term impact of physical strain on pain demonstrated in the author's previous study [6]. For each potential stressor (e.g., step count, keystrokes, number of heartbeats, calories, time spent on computer or phone), three variables were calculated. (i) Acute load is the short-term rolling average representing recent exposure to a specific stressor (window range: 1-19 days). (ii) Chronic load is a longer-term rolling average representing the historical baseline exposure to that stressor (window range: 21-49 days). (iii) The ACWR is the ratio of the acute load to the chronic load, capturing sudden spikes in activity relative to the longer-term average.

The coupled formulation of ACWR used here has itself been criticized in the sports medicine literature. Because chronic load is computed over a window that overlaps with the acute window, the two are mathematically related by construction; this can produce a correlation between the ratio and an outcome independent of any true underlying physiological relationship [20]. These and related concerns have led some authors to argue against relying on the coupled ACWR as a standalone prognostic tool [20]. 

Two considerations partly, though not fully, address this concern in the present pipeline. First, acute load and chronic load were also included as separate model inputs alongside the ratio itself, so the random forest models were not restricted to the coupled ratio alone, and feature pruning had the opportunity to favor the uncoupled components had they carried more signal. Second, this study's use of ACWR is exploratory rather than a claim of established prognostic validity.

ML architecture

Separate predictive models were developed for each distinct pain site (face, knee, arm) to account for site-specific stressors (e.g., keystrokes for arm pain vs. steps for knee pain). The modeling pipeline consisted of the following four stages.

Target Definition

The prediction target was defined as the occurrence of high pain for a specific day. Similar to what was done in the previously mentioned chronic pain study with co-occurring opioid use disorder [10], high pain was defined dynamically as a pain score exceeding a percentile threshold of the training period's pain distribution. The specific percentile used (70th, 80th, 90th, or 95th) was treated as a hyperparameter, selected via the grid search described below. For any given percentile choice, the threshold value itself was computed exclusively from training-period data, never from validation or test data. This relative thresholding ensures the model adapts to the patient's baseline severity (as different pain sites could have different baselines).

Model Selection and Training

A random forest classifier (ensemble learning) was utilized, selected for its capacity to handle non-linear interactions between physiological features as well as its inherent regularization mechanisms (specifically bootstrap aggregating and random feature subsetting), which effectively reduce model variance and mitigate the risk of overfitting [21]. The model was configured with 200 estimators and a maximum depth of five to prevent overfitting. To address the inherent class imbalance (as high-pain days are less frequent than non-flare days), balanced class weighting was applied. 

Data Inputs

The random forest classifier utilized the derived features (acute load, chronic load, and ACWR value) as inputs, crucially incorporating the data from the target prediction day. Because the daily pain score is logged each evening, after that day's activities have already taken place, no predictor value used here was recorded after the pain outcome it predicts: this is a same-day, concurrent classification, not a look-ahead one. Because the model is designed for prospective load management, a real-world deployment would require the user to estimate and input their planned activities for the day (e.g., each morning), allowing the algorithm to generate a preemptive symptom flare prediction based on that intended workload.

Feature Selection and Hyperparameter Optimization

A grid search approach was employed to optimize hyperparameters for each pain location. First, feature pruning: the initial random forest model was trained on the full set of candidate variables (all chronic loads, acute loads, and ACWR values for each stressor). Feature importance was calculated (determined by the mean decrease in Gini impurity), and a second model was then retrained using only the top k features (where k∈{5,10}) identified in the first trained model, with k being another hyperparameter of the model (see below). This pruning-and-retraining step was repeated independently for every hyperparameter combination, using only training data; the validation and test sets were never seen during feature selection.

Second is the window optimization, where a grid search approach tested all combinations of hyperparameters for the following values. Acute rolling window: 1, 3, 5, 7, 9, 11, 13, 15, 17, 19; Chronic rolling window: 21, 28, 35, 42, 49; Number of features pruning k: 5, 10; Percentile for high pain detection: 0.70, 0.80, 0.90, 0.95. The results for each hyperparameter combination were compared using two successive evaluation metrics.

Evaluation metric at the body region ML model level

The aim was to provide a clear separation between low- and high-pain days. To do so, daily high pain prediction probabilities (P), obtained after running inferences on a dataset of multiple successive days, were divided based on the ground truth pain Y score using high ($q_{\mathrm{high}} = 0.95​$) and low ($q_{\mathrm{low}} = 0.90​$) quantile thresholds. High pain ($G_{high}​$): days where $Y(t) > \mathrm{Quantile}(Y, q_{high})​$. Low pain clean ($G_{low\_clean}​$): days on which current pain and the maximum pain over the preceding 5-day window remain $\le \mathrm{Quantile}(Y, q_{low})​$. Days falling between these thresholds, or days with low current pain but elevated pain in the preceding five-day window, were classified as an intermediate buffer group and excluded from the binary discrimination objective to ensure clear separation between distinct flare and non-flare states. The evaluation metric used is the receiver operating characteristic (ROC)-area under the curve (AUC) of the predicted risk probabilities between the $G_{high}​$ and the ​$G_{low\_clean}​$ groups. Results over 0.5 indicate the model successfully assigns higher prediction probabilities (P) of risk to high-pain days compared to stable low-pain clean days.

Composite global objective function at the hyperparameter combination level

To evaluate the efficacy of hyperparameter configurations across groups of predictive models (each group containing one face, one knee, and one arm pain ML model), a composite global objective function, denoted as the adjusted score, was employed. For any given combination of hyperparameters, the algorithm first calculates the average ROC-AUC achieved by each specific pain model, referred to as the unadjusted score. The adjusted score is then computed as the mean of these individual scores minus their sample standard deviation. Subtracting the standard deviation from the mean acts as a lower-bound performance estimate, with a one-standard-deviation multiplier chosen as a simple heuristic. It ensures that the selected hyperparameters do not simply overfit to one particular pain type at the expense of the others. Instead, the metric favors stable, generalizable configurations that deliver reliably strong discrimination across all evaluated body regions.

Model training, evaluation, and actionable visualization prototype (the 'traffic light' system) 

The new dataset (and then the old dataset, in a second experiment) was divided into three sequential subsets: the first 75% of the data was the training + validation set, whereas the last 25% of the data corresponded to the testing set. The data was divided strictly according to chronological order, and no future observations were used during training or validation. Within the training + validation set, the first 70% was the training set, whereas the last 30% was the validation set (for the old dataset, a 50/50 split was used).

Random forest models were trained on the training dataset using the previously mentioned hyperparameter grid, evaluating the results on the validation set via the global composite evaluation metric. The top 15 hyperparameter sets were selected from these evaluations, using only the validation data, and the test set played no role in this selection. The choice of 15 hyperparameters is a heuristic choice. These optimal configurations were then used to retrain 15 separate random forest models on the merged training and validation sets. At inference time on the test set, the 15 resulting prediction probabilities were averaged to generate the final prediction probability.

To classify the daily risk of a pain flare in the test set, an adaptive 'traffic light' algorithm was implemented. This algorithm assigns a low (green), medium (orange), or high (red) risk level to each day based on the model’s predicted probability of a flare. To preserve strict temporal causality and prevent look-ahead bias, the risk thresholds for any given day *d​* are dynamically calculated using only historical data from days *0​* through *d-1​*. The first two days (*d = 0​* and *d = 1​*) are initialized as low risk (green) by default. For *d ≥ 2​*, the algorithm determines the threshold using a two-tiered approach, depending on the availability of historical pain variance.

Empirical Thresholding

If the patient's history contains both low-pain days (pain score *&lt; 3.0​*) and moderate-pain days ($3.0 \le \text{pain score} &lt; 3.3​$), the minimum predicted probability across all historical low-pain days ($P_{\min}​$) and the maximum predicted probability across all historical moderate-pain days ($P_{\max}​$) are identified. Provided there is sufficient separation between these values ($P_{\max} - P_{\min} > 0.05​$), the high-risk ($T_{\mathrm{red}}​$) and medium-risk ($T_{\mathrm{orange}}​$) thresholds are established as: $T_{\mathrm{red}} = P_{\max}​$; $T_{\mathrm{orange}} = P_{\max} - 0.1(P_{\max} - P_{\min})​$.

Distributional Thresholding (Fallback)

If the historical data lack either low-pain or moderate-pain days, or if the probability separation is insufficient (*≤ 0.05​*), the algorithm reverts to a standard distributional rule. The thresholds are calculated using the mean (*μ​*) and sample standard deviation (*σ​*) of all prior predicted probabilities up to day *d-1​*: $T_{\mathrm{orange}} = \mu + 0.5\sigma​$; $T_{\mathrm{red}} = \mu + \sigma​$.

Classification

Once the daily thresholds are established, the current day's predicted probability ($p_d​$) is evaluated. The day is classified as high risk (red) if $p_d \ge T_{\mathrm{red}}​$, medium risk (orange) if $T_{\mathrm{orange}} \le p_d &lt; T_{\mathrm{red}}​$, and low risk (green) otherwise. This method ensures that the warning signals remain highly tailored to the individual's evolving baseline risk.

Linear analysis (sequential Pearson correlation and residual extraction)

To identify primary and secondary stressors contributing to regional pain (face, knee, and arm) from a linear perspective, a sequential Pearson correlation and residual extraction analysis was conducted. Candidate stressors were evaluated across the following explicit temporal lag windows: same-day (lag 0), previous day (lag 1), and rolling averages over the previous two, four, 15, 30, 45, and 60 days, alongside a 15-to-30-day ACWR. For each body region, the procedure involved three iterative stages.

Selection

The stressor demonstrating the strongest significant positive correlation with the current pain variable was identified, determined by an unadjusted p-value < 0.05, used here as an exploratory ranking criterion to select each stage's leading candidate rather than as a confirmatory test of statistical significance, without correction for multiple comparisons across the numerous stressors, lag windows, and residual-removal stages considered. The resulting hierarchy of stressors is intended to generate hypotheses for future, independently powered confirmatory testing, not to establish significance in its own right.

Extraction

The variance associated with this primary stressor was removed using ordinary least squares linear regression. Specifically, a univariate linear model was fitted using the selected stressor to predict the target pain variable. Pain residuals were then mathematically isolated by subtracting these model-predicted pain values from the actual observed pain scores.

Iteration

The resulting residuals were utilized as the new dependent variable for the subsequent correlation cycle. This process was repeated for a total of three stages (one baseline evaluation followed by two successive residual removals) to establish a hierarchy of associated stressors without the linear masking effects of primary variables.

Load irregularity analysis (impact of stressor variability)

To evaluate whether load irregularity is associated with symptom exacerbation, independently of total volume, a continuous 'load irregularity index' was engineered. For each stressor, a seven-day rolling window of the absolute day-to-day differences in load, normalized by the total volume over that same period, was calculated. To prevent reverse-causation bias (e.g., resting due to pain), all predictors were lagged by one day. The index was defined as $\text{Load Irregularity Index}_t = \frac{\sum_{i=1}^{7} \vert{}L_{t-i} - L_{t-i-1}\vert{}}{\sum_{i=1}^{7} L_{t-i}}​$, where $L_t​$ is the load on day *t​*. After excluding seven-day windows with zero total volume, a Pearson correlation analysis was conducted to compare both the lagged seven-day volume and the lagged load irregularity index against the raw daily pain score (*p &lt; 0.05​*).

Statistical software

Statistical analyses were performed in Python version 3.12.8 (Python Software Foundation, Wilmington, DE, USA). Data handling and numerical computations were carried out using pandas version 2.2.3 and NumPy version 2.3.3. Random forest classification models were built using scikit-learn version 1.7.1, which was also used for ROC curve computation and, separately, for rescaling pain intensity onto a comparable 0-1 range for the traffic-light figures. This plotting-only rescaling was not applied to any model input feature. Pearson correlation analyses were performed using SciPy version 1.15.2. Figures were generated using Matplotlib version 3.10.1. The code and data used for this study were open-sourced at https://github.com/oliviermirat/MyAIGuide/tree/master/2026firstAICoachPrototype on GitHub. 

Reproducibility

All random forest models in this pipeline, both the feature-pruning pre-model and the final classifiers, were trained with a fixed random seed (random_state = 42), and no other source of randomness (e.g., random train/validation/test splitting) was used. The code and data are additionally archived at https://github.com/oliviermirat/MyAIGuide/tree/941a96d5784b516b5cf5d026e831122138c0d4cf for reproducibility purposes. Objective data were obtained from the Garmin Vivosmart 5 via Garmin Connect's bulk data export feature (a web-based request producing a downloadable ZIP archive), rather than a live application programming interface. The device's firmware was kept continuously updated to the latest available version throughout the study period rather than fixed at a single version. On days where computer mouse/keyboard click counts (tracked via WhatPulse) were missing but computer usage time (tracked via ManicTime) was recorded, click counts were estimated by multiplying that day's computer time by the participant's historical average ratio of clicks to computer time. A single day's pain score (October 7, 2023) was missing and was imputed by carrying forward the previous day's recorded value.

## Results

Personalized pathophysiological hypotheses

Pearson Correlation Coefficients Through Successive Residual Removal

Table 1 summarizes the results of the Pearson-based linear analysis on the new dataset, detailing the top positively correlated stressors for each body region across the successive residual removal stages. As detailed in the table below, the strongest correlates for regional pain consistently involve stressors that place direct mechanical or static loads on the respective anatomical regions. For example, extensive computer use and riding in a moving vehicle require sustained, static contractions of the facial and ocular musculature. Similarly, moderate-to-high-intensity cycling and walking involve repetitive knee flexion, while surfing (paddling), typing, and cycling (gripping handlebars) exert direct dynamic or static demands on the upper extremities. Notably, the appearance of 'cycling cumulative bpm > 110' as a trigger for face pain is likely a spurious correlation, driven by its high covariance with 'time spent on computer' (e.g., 0.14 Pearson correlation over a 15-day rolling window, p < .001) within this specific dataset.

**Table 1:** Top correlated stressors for pain across body regions during sequential residual extraction on the new dataset The 'pre-residual' step identifies the stressor with the strongest significant positive correlation to the raw pain score for each respective body region. Subsequent 'residual removal' steps display the top correlated stressor after linearly regressing out the effects of the stressor(s) identified in the preceding step(s). 'Time window' values give the rolling-average window (in days) applied to the stressor, except where noted as an ACWR, given as '(acute window)/(chronic window)-day ACWR' (see Data Preprocessing and Feature Engineering). 'Cumulative bpm > 110' refers to the sum of (heart rate −  110) taken across every heart-rate monitoring sample recorded during the named activity that day, counting only samples where heart rate exceeded 110 bpm (samples at or below 110 contribute zero). N = 850 day-pairs contributed to each correlation calculation. ACWR: Acute:chronic workload ratio

Body region	Residual removal step	Stressor identified	Time window identified	Pearson correlation coefficient	p-value
Face	Pre-residual	Time spent on computer	60 days	0.45	< 0.001
Face	Step 1	Time spent riding in a vehicle	60 days	0.29	< 0.001
Face	Step 2	Cycling cumulative bpm > 110	60 days	0.34	< 0.001
Knee	Pre-residual	Cycling cumulative bpm > 110	60 days	0.22	< 0.001
Knee	Step 1	Number of steps taken	15/30-day ACWR	0.10	0.003
Knee	Step 2	Sleep score	15/30-day ACWR	0.11	0.001
Arm	Pre-residual	Surf cumulative bpm > 110	15 days	0.29	< 0.001
Arm	Step 1	Cycling cumulative bpm > 110	30 days	0.30	< 0.001
Arm	Step 2	Number of keyboard and mouse clicks	1 day	0.15	< 0.001

In contrast, systemic physiological and psychological variables rarely emerge as primary drivers of regional pain. The sleep score appears only once in the positive correlation hierarchy (during the second residual removal) and only once when the analysis is inverted to evaluate negative correlations (arm pain, first residual removal). To verify whether sleep score’s single appearance was meaningful, both statistical probability and biological relevance were considered. To assess this statistically, each region's three residual-removal steps (Table 1) were modeled as drawing three winning stressors, without replacement, from the same pool of 12 candidates. Because a stressor's variance is regressed out once it wins a step, it cannot win again within the same region; this gives any specific stressor a three-in-12 (25%) chance of winning within a given region. Treating the three regions as independent, the number of the nine total slots won by a specific stressor by chance alone follows a binomial (3, 0.25) distribution, confirmed by direct simulation. Under this model, the probability of winning exactly one slot, sleep score's observed count, is 42%, and the probability of winning at least one slot is 58% (one-sided). However, unlike the localized mechanical stressors, which logically cluster in the specific anatomical regions they physically load (e.g., step count for the knee, keyboard clicks for the arm), sleep score is a systemic variable with no localized mechanism. Therefore, its single, isolated appearance is entirely consistent with background random chance rather than a genuine pathophysiological driver.

General mood does not appear as a top correlate in either analysis. When evaluating all statistically significant correlations (not just the top one) across all residual removal stages, 72.22% of the correlations for general mood are positive (suggesting that an elevated mood likely facilitates higher physical activity levels, subsequently driving localized pain) compared to only 27.78% being negative (which would imply that lower mood drives higher pain). Sleep score exhibits a more balanced distribution (44.44% positive, 55.56% negative). Collectively, these findings indicate that local mechanical loading clearly outweighs systemic factors like mood and sleep in driving acute regional pain.

Load Irregularity Analysis (Impact of Stressor Variability)

When evaluating the continuous load irregularity index against acute pain flares on the new dataset, the statistically significant findings (*p &lt; 0.05​*) linking load irregularity to pain were examined to identify stressors whose irregularity was most consistently associated with symptom flares. The resulting significant correlations fall into three distinct categories.

Artifacts of rest (negative correlations): several chronic, work-related postural stressors exhibited significant negative correlations with load irregularity, including phone time for arm pain (*r = -0.18​*, *p &lt; 0.001​*), phone time for face pain (*r = -0.14​*, *p &lt; 0.001​*), and computer clicks/keystrokes for face pain (*r = -0.10​*, *p = 0.004​*). Because the baseline volume for these activities is chronically high, a high load irregularity index essentially represents taking a day off. Thus, these negative correlations do not imply that erratic load is beneficial; they simply capture the pain-reducing effect of resting from a daily grind.

Spurious/proxy variables: several positive correlations emerged that likely represent spurious statistical artifacts or lifestyle bundles (e.g., travel days) rather than direct biomechanical causes. These included time spent riding in a car for arm pain (*r = 0.10​*, *p = 0.033​*) and step count for face pain (*r = 0.17​*, *p &lt; 0.001​*).

Primary mechanical correlates: the most clinically relevant findings were the positive load irregularity correlations for direct, localized mechanical loads. Specifically, surfing irregularity was significantly correlated with arm pain flares (*r = 0.21​*, *p &lt; 0.001​*), and step count irregularity was significantly correlated with knee pain flares (*r = 0.10​*, *p = 0.005​*). Load irregularity correlations were also observed for time spent driving for knee pain (*r = 0.08​*, *p = 0.034​*) and computer time for knee pain (*r = 0.07​*, *p = 0.034*). This is consistent with the findings described earlier in this study: for localized physical activities, it may not be merely the total volume of the load, but also the irregularity and sudden spiking of that load that is associated with symptom exacerbation. 

AI Model with Only One Stressor

The random forest analysis, as outlined in the methods section, was executed on the new dataset by isolating a single stressor at a time across the entire training, evaluation, and testing pipeline. All available variables were evaluated independently, including systemic metrics such as sleep score and general mood. A feature pruning parameter of *k=3​* was applied, which effectively retained the chronic load, acute load, and ACWR features for each individual stressor. For this isolated analysis, the standard ensemble approach (which normally averages predictions across 15 models utilizing the top 15 hyperparameter sets) was simplified to a single model built upon the single highest-performing hyperparameter set.

Table 2 presents the stressors ranked by their 80th-percentile ROC-AUC values. These values were calculated by combining inferences from the validation set (using models trained exclusively on the training set) and the test set (using models trained on the combined training and validation sets). Combining validation- and test-set inferences reflects one step of the continuous retraining a real deployment would undergo over time. Percentile-based ranking, rather than selecting a single hyperparameter configuration, was used to summarize performance across the full grid without requiring an arbitrary choice of which configuration to report. The 80th percentile specifically is a heuristic choice: high enough to reflect strong performance, but not the maximum, which could instead reflect chance variation in a single configuration rather than consistently strong performance.

**Table 2:** Ranking of all individual stressors based on their 80th-percentile ROC-AUC scores The outcome predicted by the random forest models is the occurrence of a 'high pain' day for the relevant body region. For each stressor, ROC-AUC was computed for every hyperparameter combination evaluated in the single-stressor grid search (10 acute-window × 5 chronic-window × 4 percentile-threshold settings, with feature-pruning k fixed at 3, for 200 combinations): one value from a model trained on the training set alone and scored on the validation set, and one value from a model retrained on the combined training and validation sets and scored on the held-out test set. These 200 validation-set and 200 test-set values were concatenated into a single pooled distribution of 400 values, and the 80th percentile of this pooled distribution is reported. Validation and test results were pooled to obtain a more stable percentile estimate, and this also reflects one step of the continuous retraining a real deployment would undergo over time: the model used for the test-set evaluation is trained on more data (training+validation) than the model used for the validation-set evaluation (training only). ROC-AUC: Receiver operating characteristic-area under the curve

Face		Knee		Arm	
Ranked stressors for face pain	80th ROC-AUC	Ranked stressors for knee pain	80th ROC-AUC	Ranked stressors for arm pain	80th ROC-AUC
Time spent on computer	0.88	Cycling cumulative bpm > 110	0.79	Surf cumulative bpm > 110	0.86
Sleep score	0.83	Number of steps taken	0.75	Upper-body cumulative bpm > 110	0.83
Number of keyboard and mouse clicks	0.83	Number of keyboard and mouse clicks	0.71	Rock climbing maximum route grade	0.8
Number of steps taken	0.76	Time spent using a mobile phone	0.68	Number of steps taken	0.75
Time spent driving a car	0.73	Time spent driving a car	0.66	Time spent using a mobile phone	0.74
Upper-body cumulative bpm > 110	0.71	Sleep score	0.65	Time spent driving a car	0.73
General mood	0.7	Upper-body cumulative bpm > 110	0.61	Time spent riding a vehicle	0.64
Cycling cumulative bpm > 110	0.68	Surf cumulative bpm > 110	0.59	Sleep score	0.63
Time spent using a mobile phone	0.64	General mood	0.57	Number of keyboard and mouse clicks	0.58
Surf cumulative bpm > 110	0.61	Time spent on computer	0.56	General mood	0.56
Rock climbing maximum route grade	0.56	Time spent riding a vehicle	0.5	Cycling cumulative bpm > 110	0.54
Time spent riding a vehicle	0.55	Rock climbing maximum route grade	0.49	Time spent on computer	0.49

Consistent with the findings from the linear Pearson correlation analysis, the results indicate that the strongest predictors of knee and arm pain are stressors that apply direct mechanical load to the specific anatomical region. In contrast, systemic variables such as sleep score and general mood are notably less prominent among the top-ranked predictive stressors. To verify this, all 12 variables were ranked by their average predictive performance (mean ROC-AUC) across the three body regions. Sleep score placed fifth overall, while general mood placed 11th. A one-sided Mann-Whitney U test, comparing each systemic variable's three region-level values against those of the 10 mechanical stressors, found no evidence that either performed better (p = 0.37 for sleep; p = 0.84 for mood); given the small number of region-level values (n = 3) feeding each comparison, this is consistent with the absence of a predictive advantage rather than a confirmed finding.

The Importance of Cumulative BPM Above 110 for Cycling

When re-evaluating the single-stressor AI model by incorporating the 'number of cycling calories,' the model yielded an 80th-percentile ROC-AUC of 0.68. This strongly contrasts with the 80th-percentile ROC-AUC of 0.79 achieved by the 'Cycling cumulative bpm > 110' metric. Similarly, integrating the 'number of cycling calories' into the sequential residual extraction (Pearson correlation) analysis left the original hierarchy unchanged; 'Cycling cumulative bpm > 110' remained a top correlate, whereas cycling calories did not emerge as a significant predictor. Furthermore, Figure 1 illustrates the divergent correlations of these two metrics with knee pain. For instance, the first and third peaks in cycling calories coincide with the highest values of 'cycling cumulative bpm > 110' and directly precede substantial spikes in knee pain. Conversely, the second and fifth peaks in cycling calories correspond to the lowest values of 'cycling cumulative bpm > 110' and result in minimal knee pain exacerbation. 'Cycling cumulative bpm > 110' is hypothesized to serve as an effective proxy for sustained, high-intensity cycling. During such periods, the combination of a high degree of knee flexion and greater force application likely poses a more significant biomechanical challenge to the joint compared to lower-intensity cycling, where comparable flexion occurs but with substantially less applied force.

**Figure 1:**
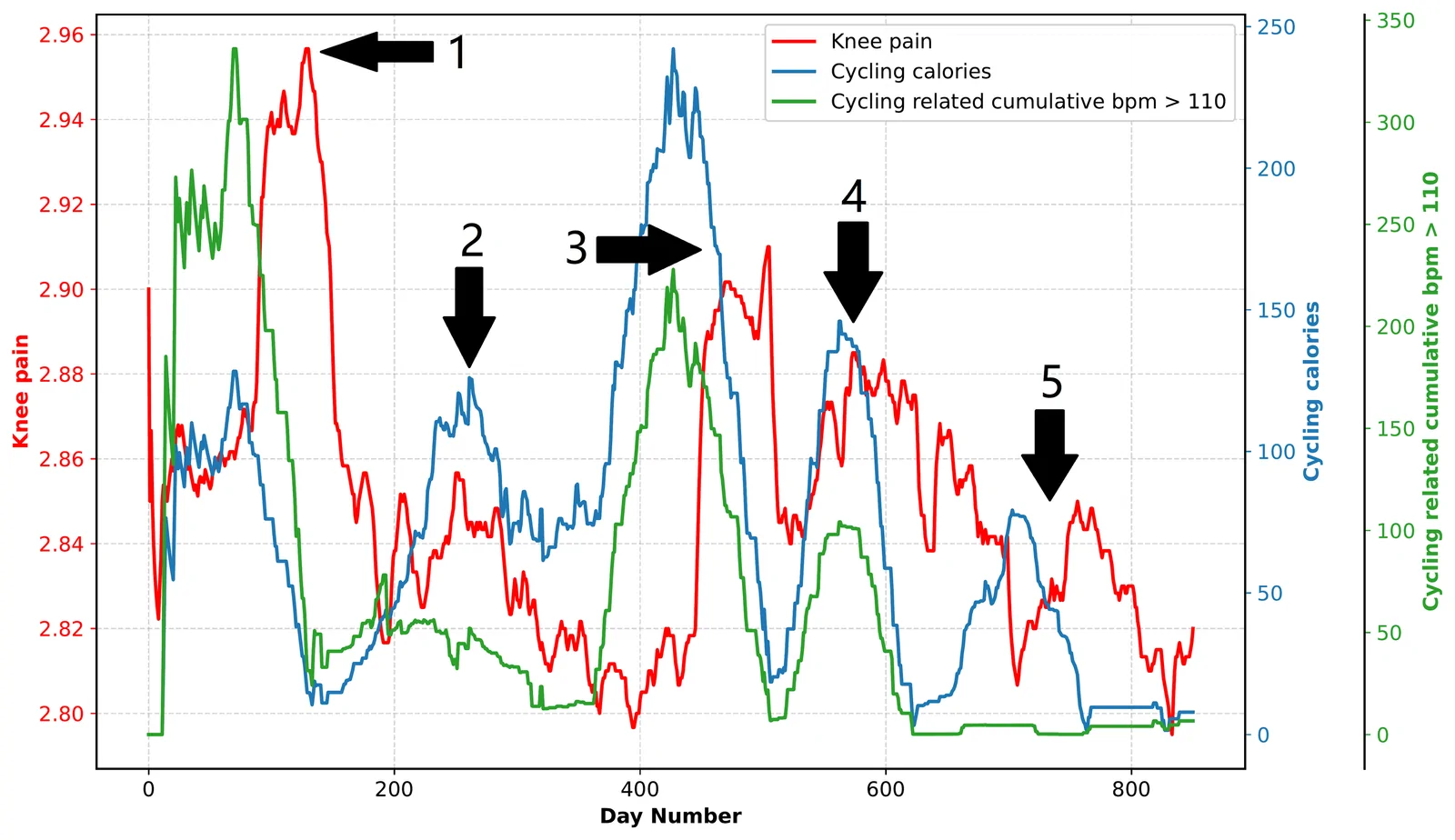
Longitudinal comparison of cycling-related physiological stressors and knee pain intensity within the new dataset A 60-day rolling average was applied to all three variables (cumulative heart rate > 110 bpm during cycling, total cycling calories, and subjective knee pain) to illustrate long-term trends. Cycling calories are Garmin’s estimated active energy expenditure from cycling (kcal/day, net of resting metabolic rate), shown as a 60-day rolling mean. Peaks 1 through 5 mark successive local maxima in the cycling calories curve, in chronological order, and are referenced in the accompanying text ('The Importance of Cumulative bpm Above 110 for Cycling'). Knee pain was rated using the custom pain scale described in Methods (Data acquisition and processing, Subjective measures).

AI health coach prototype

Baseline Analysis on the New Dataset

Figure 2 illustrates the simulated prospective 'traffic light' warnings and associated predictions on the unseen test set (from the new dataset only) using an ensemble approach averaging the risk predictions across the top 15 models. The unseen test set featured one single most severe flare for each body region, and the system successfully issued a 'red light' (for face and arm pain) or an 'orange light' (for knee pain) warning before the peak flare day for each pain type, all while issuing red warnings on 17.7%, 12.2%, and 20.1% and orange warnings on 7.9%, 5.5%, and 1.8% of days for the face, knee, and arm pain, respectively.

**Figure 2:**
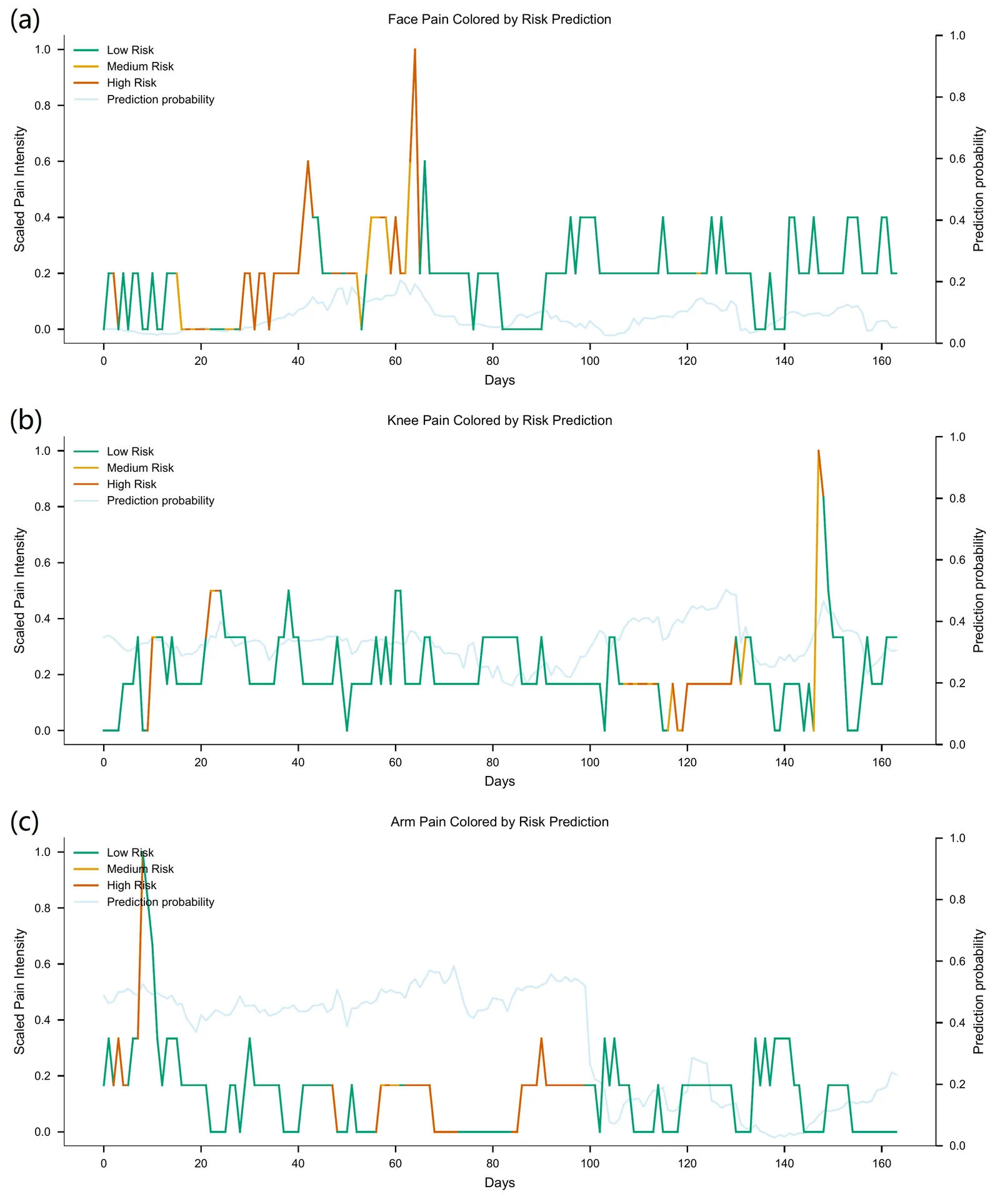
Prospective ‘traffic light’ symptom flare risk predictions across three anatomical regions (a) Face pain; (b) Knee pain; (c) Arm pain Daily flare risk predictions (Green = Low risk, Orange = Medium risk, Red = High risk) were evaluated on the unseen test set of the new dataset. The test set was strictly excluded from all training and hyperparameter optimization phases prior to inference to ensure an unbiased evaluation.

Beyond the single most extreme flare per region described above, the test set contained several other days on which pain exceeded the G_high threshold defined in the methods, with G_low_clean days again serving as the negative class. These G_high days often occurred consecutively or in close succession rather than in isolation: once pain crossed the threshold, it tended to remain elevated for several days before returning to baseline. The system is intended to alert the patient before or at the onset of a flare, not to confirm one already in progress, so scoring every G_high day belonging to the same flare as a separate detection would not reflect this goal. Patient activity also typically decreases once a flare has begun, which could alter the very stressor values used as predictors on subsequent days. For both reasons, only the first G_high day following a gap of more than seven non-flare days was scored as a target event; any further G_high day within seven days of the previous one was treated as part of the same event and excluded, like the existing buffer days. Sensitivity, specificity, PPV (Positive Predictive Value), and NPV (Negative Predictive Value) were then computed for the discrete traffic-light output on this basis. Two positive-warning criteria were used: a day counted as positive if it received an orange-or-red warning, or, more strictly, only if it received red; results below are reported as orange-or-red/red-only throughout. Under this scoring, sensitivity was 1.00/0.50 for face (two flare days), 0.50/0.25 for knee (four flare days), and 1.00/1.00 for arm (one flare day); specificity was 0.75/0.83 for face, 0.82/0.87 for knee, and 0.77/0.79 for arm; PPV was 0.05/0.04 for face, 0.08/0.06 for knee, and 0.03/0.03 for arm; and NPV was 1.00/0.99 for face, 0.98/0.98 for knee, and 1.00/1.00 for arm. Given the small number of flare days per region, these figures, particularly for arm pain (n = 1), should be read as indicative rather than precise.

Importantly, many of the prediction probability spikes that resulted in red and orange warnings, while not corresponding to an actual pain flare, did correspond to pain increases. Finally, the Pearson correlation coefficient between the validation and test set ROC-AUC scores was moderately high (r = 0.56; Table 3, Figure 3). This suggests that the validation set served as a reliable proxy for unseen data during hyperparameter tuning, as model configurations yielding higher performance during validation generally translated to higher predictive accuracy on the test set.

**Table 3:** ROC-AUC scores and Pearson correlations across dataset configurations Comparison of ROC-AUC scores on the hold-out test set for the ensemble top 15 hyperparameters selected via grid search (inference on test set); and maximum, 80th, and 50th percentiles across all sets of hyperparameters considered during the grid search (inference on validation set). Additionally, the table presents the Pearson correlation coefficients between the validation and test set (not ensemble) ROC-AUC scores across all hyperparameter sets, illustrating the stability and generalizability of the models across different parameter sets. These two groups of rows are not directly comparable: the ensemble top-15 rows report a single ROC-AUC per region from one averaged model (the top 15 configurations retrained and combined, as described in Methods), whereas the max/80th/50th rows summarize the spread of ROC-AUC values across every individual configuration in the full hyperparameter grid, with no averaging or retraining step of their own. 'Baseline' refers to the standard modeling pipeline described in the Methods section, using body-region-specific stressors at full data resolution. 'All stressors combined' refers to the same pipeline re-run using an aggregated set of all stressors across all body regions rather than region-specific isolation. 'Low resolution' and 'very low resolution' refer to artificially degraded 'baseline' stressor data, as described in Methods. ROC-AUC: Receiver operating characteristic-area under the curve

Metric		New dataset				Old dataset
		Baseline	All stressors combined	Low resolution	Very low resolution	Baseline
Inference with ensemble top 15 sets of hyperparameters	Face ROC-AUC	0.93	0.78	0.93	0.9	0.97
	Knee ROC-AUC	0.78	0.6	0.73	0.7	0.53
	Arm ROC-AUC	0.82	0.99	0.6	0.47	0.01
	Mean ROC-AUC	0.85	0.79	0.75	0.69	0.5
All sets of hyperparameters considered during the grid search	Max ROC-AUC	0.89	0.71	0.87	0.79	0.71
	80th ROC-AUC	0.74	0.57	0.69	0.67	0.60
	50th ROC-AUC	0.64	0.51	0.59	0.58	0.54
	Pearson coefficient	0.56	0.27	0.43	0.18	0.21

**Figure 3:**
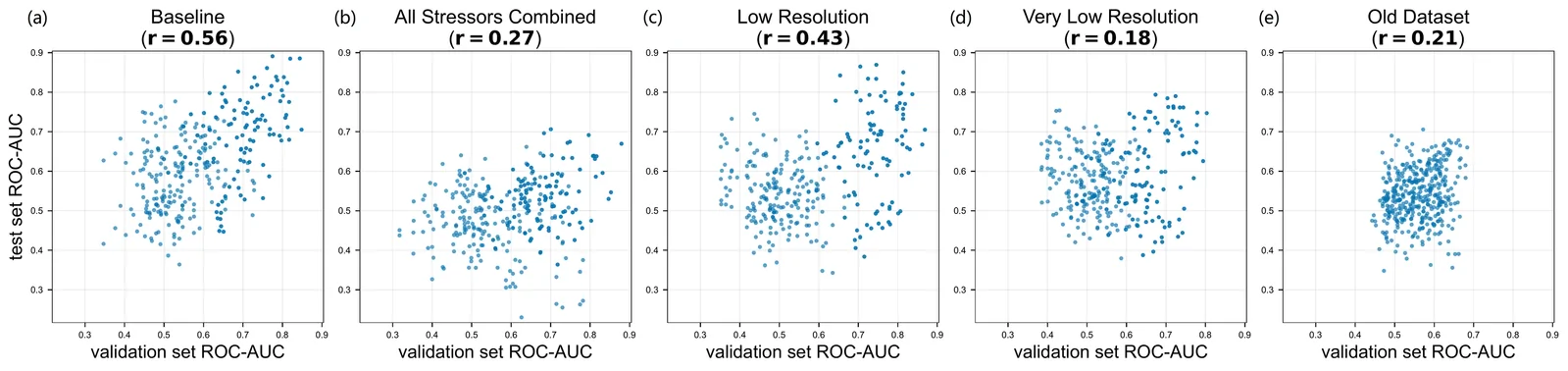
Impact of data resolution and stressor configurations on model generalizability (a) Baseline (r = 0.563); (b) All stressors combined (r = 0.270); (c) Low resolution (r = 0.434); (d) Very low resolution (r = 0.177); (e) Old dataset (r = 0.212) Comparison of ROC-AUC scores and Pearson correlation coefficients between the validation and test sets, evaluating model robustness across multiple experimental conditions on the new dataset (a-d) and on the old dataset (e), demonstrating the critical impact of both high-resolution tracking and domain-informed feature selection on predictive accuracy. The p-value is < 0.001 and n = 400 for all panels ROC-AUC: Receiver operating characteristic-area under the curve

Stressor Quality and Selection Dictate Traffic Light Performance

To evaluate the impact of feature selection on predictive performance, the pipeline was re-run without isolating body-region-specific stressors, instead supplying the random forest models with an aggregated set of all variables previously used separately for different body regions. Under these conditions, both the overall ROC-AUC scores and the Pearson correlation coefficient between the validation and test sets substantially deteriorated (e.g., from an 80th percentile ROC-AUC of 0.74 at baseline to 0.57 for all stressors combined) (Table 3, Figure 3). This highlights a limitation of the current model architecture and underscores the critical role of domain knowledge in feature engineering, demonstrating that curating physiologically relevant variables yields superior performance compared to relying solely on the algorithm for feature selection.

Next, the importance of data resolution was assessed by artificially degrading the quality of the stressors. This was achieved by removing specific variables (time spent on phone and in transit), discretizing the rock climbing maximum route grade into a binary variable (climbed on that day vs. not), and discretizing continuous cumulative heartbeats variables into three broad categorical ranks (and into two categories only for the very low resolution experiment). Reduced data resolution had contrasting effects on the tuning process and on achievable model performance. The correlation between validation- and test-set ROC-AUC across the hyperparameter grid declined substantially (r = 0.56 to 0.43 to 0.18), indicating that a configuration's validation performance became a progressively less reliable indicator of its performance on test data, a direct loss of generalizability in the model-selection process. The achievable performance ceiling, however, was less affected: the 80th-percentile ROC-AUC across the grid declined only modestly (0.74 to 0.69 to 0.67), showing that strong-performing configurations remained available at each resolution level, even as validation became progressively less able to identify which configuration would be strong. The ensemble approach actually used for the traffic-light system (Table 3, 'mean ROC-AUC') showed a larger decline (0.85 to 0.75 to 0.69), suggesting the deployed selection procedure was more sensitive to reduced resolution than the grid's best achievable performance alone would imply.

Old Dataset

The 'traffic light' analysis was repeated using the old dataset, which relied on retrospective data characterized by less granular activity logging. The model's overall ROC-AUC scores and the Pearson correlation between the validation and test sets substantially deteriorated compared to the baseline established by the new dataset (Table 3, Figure 3); however, this decline was highly uneven across regions (face: 0.93 to 0.97; knee: 0.78 to 0.53; arm: 0.82 to 0.01). This unevenness is consistent with each region's leading stressor identified in the new dataset analysis (Tables 1-2): face pain's top predictor, time spent on a computer, was tracked with comparable precision in the old dataset, whereas knee and arm pain's top predictors, cycling cumulative bpm > 110 and surf cumulative bpm > 110, respectively, could not be meaningfully reconstructed from the old dataset, which relied on artifactual step counts for cycling and a binary surfing flag rather than direct heart-rate measurement (see Data acquisition and processing). Alternatively, the degradation could stem from feature selection: as the time period and recording methods differ significantly between the two datasets, a customized, period-specific choice of stressors might have led to better results. Nevertheless, the machine learning pipeline still produced strong test-set performance for face pain (ROC-AUC = 0.97) using the optimal ensemble model, though performance across the full hyperparameter grid was more modest, with maximum and 80th-percentile test ROC-AUCs of 0.71 and 0.60, respectively, when applied to this previous dataset.

Longer-Term Analysis on the New Dataset

The primary development of the random forest AI coach prototype was conducted between January and March 2026, utilizing the new dataset exclusively. Subsequently, in July and August 2026, the model's longitudinal robustness was evaluated by applying the previously trained model to the unseen follow-up dataset. Figure 4 illustrates the model's performance across both the test portion of the new dataset (days 0 through 163) and the follow-up dataset (day 164 onward) for knee and arm pain. During the follow-up period, the most severe symptomatic peaks for both knee and arm pain failed to trigger a definitive red or orange traffic light warning. However, the algorithm's predicted probabilities for a flare did exhibit a noticeable increase prior to these events. 

**Figure 4:**
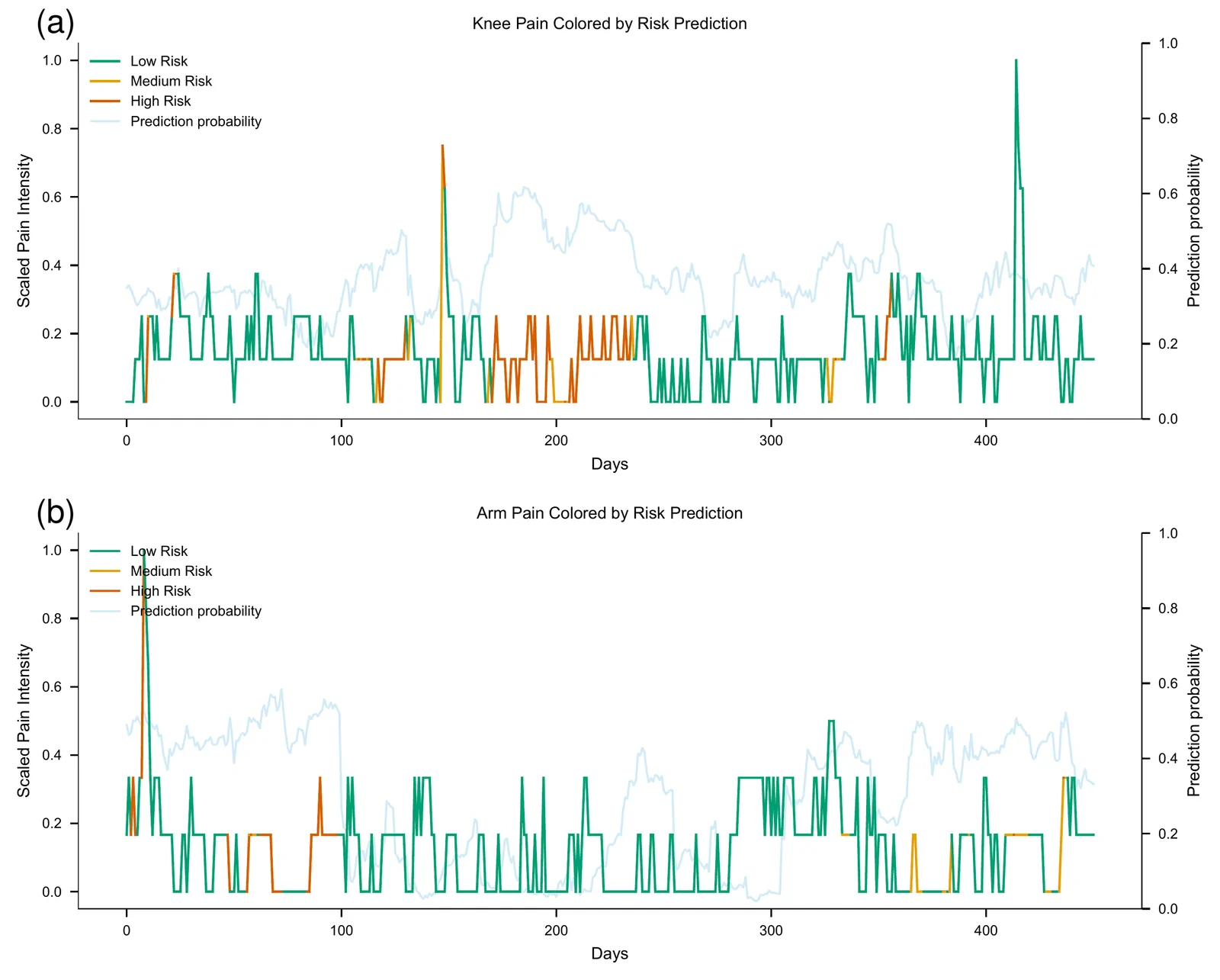
Knee and arm pain prediction across the new dataset test period and the subsequent follow-up dataset (a) Knee pain; (b) Arm pain Risk color coding and prediction probability follow the same conventions as Figure 2 (Green = Low risk, Orange = Medium risk, Red = High risk), and both figures use the identical model, trained exclusively on the new dataset (training+validation sets), with no retraining on follow-up data. Unlike Figure 2, which covers the new dataset test set only (days zero-163) for all three body regions, this figure extends the knee and arm panels through the follow-up dataset (day 164 onward).

Quantitatively, using the same flare-event grouping described above, the test set extended through follow-up contained eight flare events for knee (versus four in the test set alone) and two for arm (versus one). Sensitivity was 0.50/0.25 in the test set alone versus 0.38/0.25 in the extended window for knee (orange-or-red declining, red-only unchanged) and 1.00/1.00 versus 0.50/0.50 for arm (declining under both criteria); specificity was 0.82/0.87 versus 0.74/0.80 for knee (declining under both) and 0.77/0.79 versus 0.85/0.92 for arm (improving under both). This is broadly consistent with the visually apparent decline in detection performance described above, though not uniformly so. Given the small number of events involved, particularly for the arm, these figures should be read as suggestive rather than precise.

These mixed results underscore a well-documented challenge in medical machine learning translation: models that demonstrate robust predictive accuracy during controlled, retrospective validation often experience performance degradation when deployed prospectively. This performance gap highlights the inherent complexities of adapting laboratory-grade algorithms to the dynamic, unpredictable nature of real-world data streams [22,23]. This gap may also reflect genuine instability in the learned stressor-pain relationships over time within this individual.

Using the AI Health Coach as a Way to Evaluate Interventions

To mitigate face pain, participant #1 implemented an ergonomic intervention by transitioning from a standard liquid crystal display (LCD) monitor to a 13.3-inch DASUNG monochromatic e-ink monitor (DASUNG Tech Co., Ltd., Beijing, CHN). As illustrated in Figure 5a, following the adoption of the e-ink monitor on day 214, the baseline random forest model severely overestimated the subsequent risk of symptom flares, generating persistent high-risk (red) warnings that did not clinically materialize. However, retrospectively applying a 0.7 attenuation coefficient to the 'time spent on a computer' variable from day 214 onward effectively recalibrated the predictions, yielding a significantly more accurate estimation of flare risk (Figure 5b). This coefficient was selected by manual adjustment until predictions visually matched the observed absence of flares, rather than via a formal optimization procedure. Comparing the model's daily predicted flare probability with and without the 0.7 attenuation across the 236 days following the intervention showed a significant reduction under attenuation (mean probability 0.18 vs. 0.10; paired t(235) = 7.13, p < .001; Wilcoxon W = 7705, p < .001). This confirms the coefficient produces the intended directional effect on model output; it does not, on its own, independently validate that 0.7 reflects the monitor's true biomechanical effect, since the coefficient was chosen to match an already-known outcome. While this coefficient was derived retrospectively to fit the observed absence of flares, it illustrates one potential future application of the AI system: approximating the biomechanical benefit of a lifestyle change. In a real-world management context, such a retrospective analysis could serve as an exploratory, hypothesis-generating signal for patients and clinicians to consider alongside other evidence, rather than as independent confirmation that a given intervention was responsible for the observed change.

**Figure 5:**
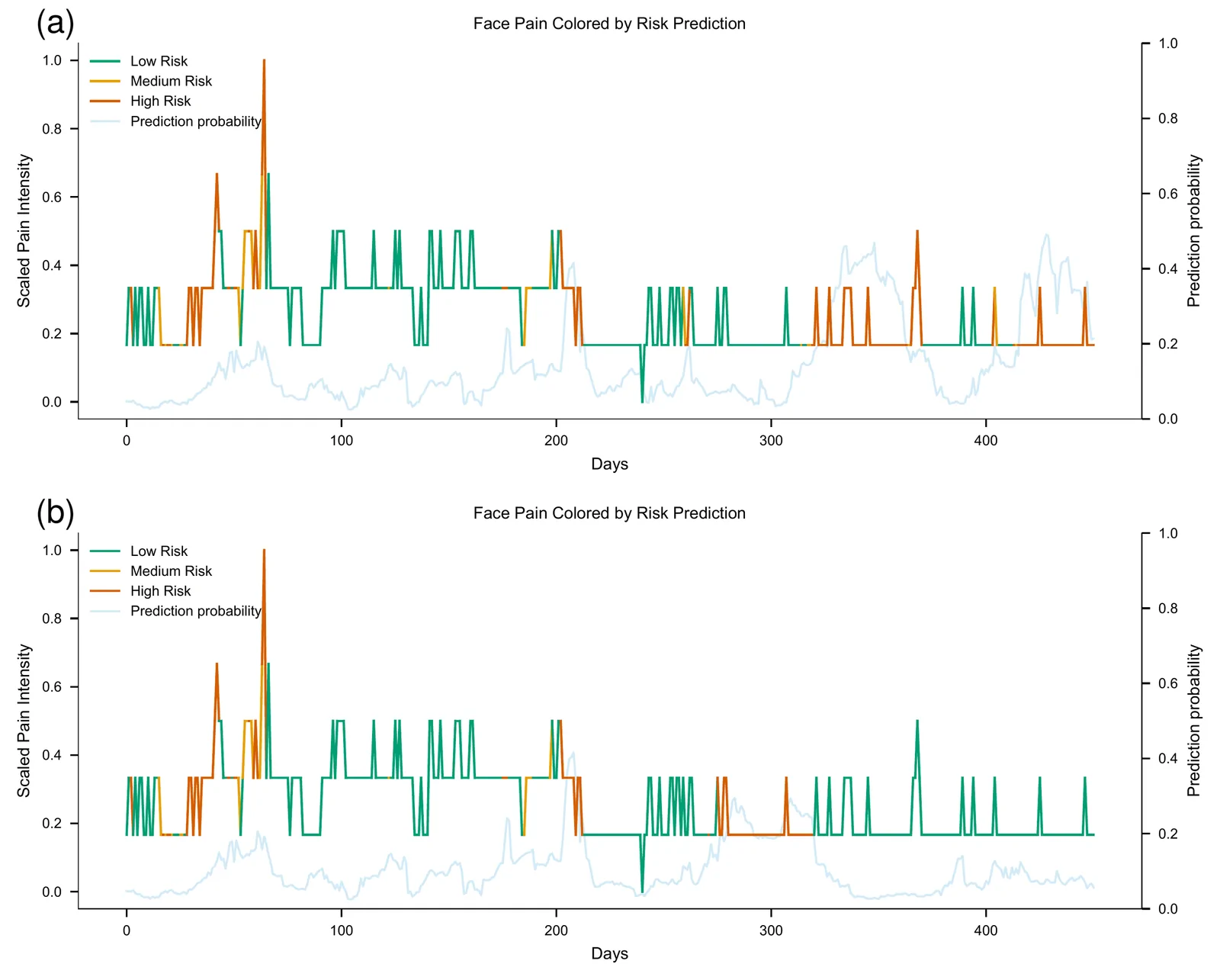
Face pain prediction before and after an ergonomic intervention The ergonomic switch was from an LCD to an e-ink monitor on day 214. Both panels apply the same model to the same data, differing only in the 'time spent on a computer' input from day 214 onward. (a) Using the actually recorded, unadjusted values, which caused the model to persistently overestimate flare risk after the monitor switch; (b) Using those values retrospectively multiplied by an attenuation coefficient of 0.7 from day 214 onward, approximating the monitor's reduced biomechanical strain, bringing predicted risk back in line with the absence of observed flares. The graph shows a combination of the new dataset (days zero-163) and the follow-up dataset (day 164 onward).

## Discussion

Generating pathophysiological hypotheses from n-of-1 data

Central sensitization [24] is fundamentally understood through two primary mechanisms: top-down and bottom-up amplification. The top-down theory posits that central nervous system alterations are the primary driver of pain, independent of significant ongoing peripheral pathology [25]. Conversely, the bottom-up theory suggests that an excess of continuous, often covert, noxious peripheral input is strictly required to initiate and maintain the sensitization of the central nervous system, ultimately amplifying pain signals across multiple pathways [26,27].

Historically, in real-world clinical practice, when a patient presented with unexplained chronic pain affecting three or more body regions, the conclusion would have often leaned more towards top-down central sensitization [28]. However, despite the patient experiencing concurrent pain in three or more body regions, the n-of-1 data presented here are consistent with a bottom-up mechanism, though a purely top-down supraspinal defect cannot be ruled out (see below). Indeed, the n-of-1 data show that the patient's sleep quality and general mood do not appear to drive symptom flares, and that instead, the pain fluctuates directly in response to localized mechanical loading, a pattern consistent with an active peripheral source, though not exclusively so. The most appropriate therapeutic approach might thus be to try, as much as possible, to decrease the peripheral pain input for this particular patient [27]. By deliberately targeting and minimizing the peripheral nociceptive signals, if bottom-up central sensitization is indeed occurring, this approach would reduce the continuous fuel sustaining it, which could successfully diminish global pain sensitivity and prevent further central wind-up.

Since this patient does not have any visible physiological damage or overt structural abnormalities that can be seen on standard radiographs or blood tests, standard structural interventions are inapplicable. To effectively decrease the peripheral pain input, the most appropriate approach for this patient might be to follow Dye's 'envelope of function' theory [19]. The envelope of function refers to the specific boundary of load and frequency that a joint can safely tolerate without becoming irritated and signaling pain. When mechanical loads exceed this personal threshold, the peripheral tissues become stressed and initiate localized nociceptive firing, even in the absolute absence of macroscopic structural injury. By carefully managing the patient's daily activity to stay strictly within their individual envelope of function, the covert peripheral nociceptive input potentially driving their widespread central sensitization may be minimized.

An alternative mechanistic hypothesis cannot be entirely ruled out. While the data are consistent with bottom-up central sensitization driven by peripheral load, it is possible that the mechanism is actually primarily top-down. Under this alternative model, the central nervous system autonomously generates a heightened, supraspinal gain (a generalized hypersensitivity), and local mechanical load merely dictates the allocation of that pain. In this scenario, slightly elevated load in one region acts as a spotlight, drawing the nervous system's focus and manifesting the top-down sensitization locally, rather than the mechanical load acting as the primary, bottom-up fuel for the sensitization itself. Differentiating between a load-driven bottom-up mechanism and a top-down allocation model remains a complex challenge for future continuous tracking studies. However, even in that scenario, reducing mechanical load by adhering to the envelope of function theory remains a reasonable, low-risk approach to consider, as preventing pain flares associated with elevated mechanical stress may meaningfully contribute to management under either mechanism, though this has not been independently tested in this patient.

Generating personalized advice for the n-of-1 patient

Translating statistical correlations from observational data into clinical advice requires careful consideration, as these associations may be spurious or heavily dose-dependent rather than strictly causal. Identifying a specific activity as a predictive risk factor does not inherently label that activity as universally harmful. Instead, it indicates that the specific volume or dose performed during the study period crossed a threshold from a beneficial or neutral zone into a detrimental one. For example, while surfing emerged as a risk factor at the high volumes observed prior to a symptom flare, it might remain highly therapeutic at a lower, carefully managed dose. Despite these inherent uncertainties, leveraging high-density n-of-1 data allows for the formulation of practical, personalized management hypotheses. Based on the specific data patterns observed for the participant in this study and from the preceding analysis of the likely pathophysiology in the previous section, the following targeted advice may be proposed.

Cycling modifications: exercise caution regarding moderate-to-high-intensity cycling. The combination of a high knee flexion angle and significant applied force may present a biomechanical risk, potentially driving long-term vulnerability even if acute damage is not immediately apparent. Progressive load management: applying Dye’s envelope of function approach [19] to load management might be a useful therapeutic approach, though its benefit for this patient has not been independently tested. Upper-body pacing: apply a similarly progressive and consistent approach to surfing. Whether swimming would offer a comparable benefit with lower biomechanical cost is a hypothesis worth testing in future studies. Ergonomic interventions: actively mitigate static postural loads associated with computer use, given this variable's strong association with face, knee, and arm pain. The model's predictions were consistent with reduced face-pain flares following the transition to a monochromatic e-ink monitor (see Results); further ergonomic modifications targeting knee and arm positioning may be worth exploring using a similar approach.

While these recommendations cannot be delivered with absolute certainty, they provide a highly personalized, empirical starting point for behavioral and lifestyle adjustments. Generating these data-driven hypotheses can serve as a catalyst for patient self-reflection, or they can be systematically tested and validated using structured n-of-1 trial methodologies [29]. Ultimately, AI functions as a powerful adjunctive tool for clinical decision-making. However, translating predictive insights into safe therapeutic load management requires careful, collaborative interpretation by both the patient and their healthcare provider. To facilitate the widespread, real-world deployment of these systems, future research should prioritize the development of specialized software and intuitive graphical user interfaces.

AI coach proof of concept for chronic non-specific musculoskeletal pain 

This study also aimed to explore the feasibility of a prospective forecasting engine for chronic non-specific musculoskeletal pain, moving beyond retrospective population-level association studies. In alignment with the shift toward 4P medicine, a proof-of-concept n-of-1 decision support system was developed. This system translates continuous daily activity and wearable data into an actionable 'traffic light' (green/orange/red) flare warning.

The initial results are encouraging: during the unseen testing phase of the new dataset, the model successfully flagged the single most severe flare for each of the three monitored body regions, with specificity for the broader set of flare events remaining reasonably high across regions, a relevant consideration for limiting alert fatigue in real-world use, though this should be confirmed at scale. Importantly, many of the prediction probability spikes that resulted in red and orange warnings, while not corresponding to an actual pain flare, did correspond to pain increases. The shift to the higher-resolution new dataset may have been highly advantageous, resulting in improved predictive performance. Overall, these findings suggest that leveraging big data generated by wearable devices at the individual level, combined with personalized AI, has the potential to capture individualized precursors to symptom flares. This approach naturally paves the way for just-in-time adaptive interventions [13]. By processing continuous data streams from wearables in real time, such AI systems can provide timely, personalized warnings, empowering patients to proactively manage their pacing and adjust their daily routines before a flare fully manifests.

However, evaluating the model over a longer horizon using the follow-up dataset revealed the practical complexities of real-world deployment. During this follow-up period, the most severe symptomatic peaks for both knee and arm pain failed to trigger a definitive red or orange warning. Despite missing the explicit threshold alerts, the algorithm's underlying predicted probabilities for a flare still exhibited a noticeable increase prior to these events. This performance gap highlights a well-documented challenge in medical machine learning translation: models that demonstrate robust predictive accuracy during controlled validation often experience performance degradation when deployed prospectively [22,23]. However, because this evaluation involved the same participant, stressors, and modeling pipeline as the original test set, rather than a new patient population or clinical setting, this gap may also reflect genuine instability in the learned stressor-pain relationships over time within this individual.

It should also be noted that this system presents several other potential limitations. First, the current iteration of the system required substantial domain knowledge to select the most appropriate input parameters, as introducing all available variables indiscriminately reduced model efficacy. Second, predictive models are inherently susceptible to spurious correlations, and out-of-distribution (OOD) data remains a persistent risk in n-of-1 datasets, as novel activities or stressors not seen during the training phase can drastically affect prediction accuracy. Finally, beyond these methodological constraints, another subtle but critical consideration that should be noted while designing AI health coaches is the potential for a nocebo effect, where preemptive warnings of an impending flare might induce anxiety or hypervigilance, inadvertently exacerbating subjective pain.

Study limitations

Methodologically, because this study utilized a single-subject n-of-1 design, the framework must be rigorously validated across other individuals. Additionally, the ground truth relied on subjective daily pain intensity logs on a 0 to 10 scale, which were inherently vulnerable to self-reporting bias. It is also worth noting that the pain and mood scales used here were developed by and calibrated to this specific participant; unlike a standardized instrument, they cannot be independently applied or reproduced by other researchers or in other patients, which is an intrinsic limitation of this n-of-1 approach. Moreover, because the sole participant in this n-of-1 study is also the primary researcher, this creates a bidirectional risk: the same unblinded individual, aware of his own hypotheses, both logged the subjective outcome (pain, mood) and selected which stressors to track. This is a particular concern for the study's central finding that mechanical stressors outperformed systemic variables, since an a priori expectation, carried over from the participant's prior work [6], that load drives his pain could have inflated the perceived or reported coupling between load and pain relative to mood or sleep; the comparatively less precise measurement of mood and sleep relative to mechanical stressors (discussed further below) may compound this.

This unblinding also extends to the chronological train/validation/test structure used throughout this study: because the participant lived through and was informally aware of his own symptoms and activities across the entire multi-year period spanned by the old, new, and follow-up datasets, well before any formal split or modeling took place, none of the resulting splits can achieve the same degree of blinding to held-out data that a prospective design analyzed by someone without this prior exposure would provide. This is a structural feature of single-subject self-experimentation research, rather than a specific or correctable analytic choice, and applies across this study's analyses generally, distinct from, and broader than, the narrower instances already identified above as exploratory and post-hoc. Finally, the feature engineering in this study heavily depended on the ACWR framework. While a standard in sports medicine for acute injury prediction, ACWR might oversimplify the complex, multifactorial mechanisms underlying non-specific chronic pain.

One further limitation of the linear analysis in Table 1 is the reliance on unadjusted p-values within a longitudinal time-series dataset. In continuous n-of-1 tracking, daily observations are inherently not independent; pain fluctuates in waves and behaviors occur in multi-month phases, introducing strong temporal autocorrelation that is further magnified by extended rolling windows. This autocorrelation can inflate standard statistical confidence by interpreting a single prolonged lifestyle shift as dozens of independent data points. Consequently, some secondary correlations may be mathematically fragile and highly susceptible to collinearity. The unadjusted p-values should therefore be interpreted with caution, and these associations must be viewed strictly as data-driven hypotheses rather than definitive causal proofs. This same caution extends to the mechanistic and therapeutic interpretations discussed, which are ultimately grounded in these same correlations. 

Regarding random forest evaluation, because intermediate 'buffer' days between 'high pain' and 'low pain clean' days were excluded from the evaluation, the reported ROC-AUC metrics represent a somewhat idealized score that does not fully reflect model performance across ambiguous, day-to-day pain fluctuations. However, this targeted exclusion served as a necessary practical step to mitigate the inherent unreliability of subjective mid-range pain logging. By deliberately filtering out this noisy data, the approach successfully maximized the signal-to-noise ratio and ensured the algorithm could accurately identify the most clinically impactful flare events. 

Another constraint stems from the reliance on a commercial wearable device, Garmin Vivosmart 5, for objective physiological metrics, which introduces algorithmic uncertainty. Proprietary metrics, such as the daily sleep score, are 'black boxes' that may not accurately reflect true sleep architecture or systemic recovery, potentially skewing their predictive value or masking their true physiological impact. Similarly, the subjective daily logging of general mood on a 0 to 10 scale is inherently vulnerable to transient emotional states, recall bias, or day-to-day variations in the participant's interoceptive awareness, which could render these conclusions inaccurate.

Additionally, this study relied solely on global daily self-reported pain intensity; no quantitative sensory testing, validated central-sensitization screening instruments (e.g., the Central Sensitization Inventory), or other assessment of altered sensory processing was performed, nor was a controlled paradigm testing whether pain persists after peripheral input is reduced or removed. Central sensitization itself remains a broadly and inconsistently defined construct in the pain literature; regardless of which definition is applied, none of the assessment approaches currently associated with it were used here. The central sensitization hypotheses discussed above should be read with this constraint in mind. Despite these limitations, the longitudinal data remain consistent with, though do not establish, a load-related pain mechanism such as bottom-up central sensitization. Because reducing mechanical load is a reasonable, low-risk avenue to explore regardless of which mechanism is ultimately correct, utilizing the envelope of function as a therapeutic framework may still represent a practical starting point for this patient, though its clinical benefit here has not been independently tested.

Future perspectives

A key next step is to evaluate this methodology across a larger, diverse cohort of patients. Beyond verifying that the success of this n-of-1 study was not an isolated occurrence, a broader trial would provide population-level data. This global statistical baseline could be used to pre-train models that are subsequently fine-tuned to individual patients, conceptually similar to transfer learning in deep neural networks. Furthermore, the individualized hypotheses generated by the combination of AI and statistical methods could be systematically tested using n-of-1 trial frameworks [29].

A particularly exciting future perspective is the integration of biological variables as additional continuous stressors. Because the true biological causes of non-specific chronic pain remain largely unknown, expanding digital phenotyping to include biological markers and physiological metrics could drastically improve model accuracy. More ambitiously, applying advanced AI to the massive volumes of data generated by combining continuous biological metrics with lifestyle and behavioral tracking could help decode the underlying biological mechanisms of non-specific chronic pain. This could uncover hidden, objective physiological triggers that precede subjective pain flares, bridging the gap between mechanical load tracking and systemic biological responses. Ultimately, this integration could shift the field from merely managing symptoms toward achieving true causal understanding. Potential technologies to explore in this context include longitudinal tracking via 7T MRI [30] and blood transcriptomics/proteomics [31], as well as single baseline assessments like full-genome sequencing to provide broader biological context.

## Conclusions

This n-of-1 observational study demonstrates the feasibility of using continuous, high-density wearable data to generate personalized, actionable hypotheses for non-specific chronic musculoskeletal pain. By integrating linear correlation analysis with machine learning models, the findings identify a stronger association with specific, localized mechanical stressors than with systemic variables like mood or sleep quality. This is consistent with, but does not establish, a load-related pain mechanism such as bottom-up central sensitization for this specific patient, and raises the envelope of function as a candidate practical framework for daily load management, though its clinical benefit has not been independently tested. 

Furthermore, the development of a prospective artificial intelligence health coach prototype illustrates the potential for just-in-time adaptive interventions in chronic pain management. While real-world deployment challenges remain, such as performance degradation over time and the necessity of high-resolution data, the system successfully provided a proof-of-concept for personalized flare risk warnings, enabling proactive pacing and illustrating how such a system might help generate hypotheses about the impact of lifestyle interventions. Future research should expand this methodology to broader cohorts and integrate objective biological markers, ultimately bridging the gap between associative forecasting and a true causal understanding of chronic pain.
